# Supplementary material for: Development and Validation of Targeted Gene Sequencing Panel Based Companion Diagnostic for Korean Patients with Solid Tumors
Source: Cancers (Basel). 2021 Oct 12;13(20):5112. doi: 10.3390/cancers13205112 (PMC8534153; doi:10.3390/cancers13205112)
Supplement: Supplementary file 1 [file cancers-13-05112-s001.zip › Supplementary Table S1.pdf]

Supplementary Table S1. Specificity of Korean CDX panel results with KRAS direct sequencing samples.

KRAS variant status	Sample ID	Variant calling	Variant Statistics		Sample Statistics	
			Alt count*	Ref count*	Mean Depth	On target cover rate
**Wild type**						
	FFPE 1	Negative			1186.0	0.99
	FFPE 4	Negative			786.6	0.99
	FFPE 6	Negative			2050.3	0.99
	FFPE 7	Negative			1220.7	0.99
	FFPE 8	Negative			2848.7	0.99
	FFPE 10	Negative			2500.4	0.99
	FFPE 11	Negative			1451.7	0.99
	FFPE 24	Negative			1427.0	0.99
	FFPE 26	Negative			1165.4	0.99
	FFPE 27	Negative			594.7	0.99
	FFPE 33	Negative			589.2	0.99
	FFPE 34	Negative			1979.6	0.99
	FFPE 37	Negative			1112.3	0.99
	FFPE 38	Negative			2406.2	0.99
	FFPE 64	Negative			1530.6	0.99
	FFPE 68	Negative			1066.9	0.99
	FFPE 69	Negative			1136.6	0.99
	FFPE 70	Negative			2012.7	0.98
	FFPE 76	Negative			1659.9	0.99
	FFPE 78	Negative			1332.9	0.99
	FFPE 79	Negative			1497.8	0.99
	FFPE 86	Negative			1841.1	0.99
	FFPE 88	Negative			1220.1	0.99
	FFPE 96	Negative			1686.3	0.99
	FFPE 97	Negative			2014.6	0.99
	FFPE 99	Negative			2913.1	0.99
	FFPE 103	Negative			1936.9	0.99
	FFPE 105	Negative			2922.0	1.00
	FFPE 117	Negative			3431.9	0.99
	FFPE 125	Negative			3176.0	0.99
	FFPE 21	Positive (Gly13Asp)	164	1,271	929.5	0.99
	FFPE 28	Positive (Gly12Asp)	45	524	801.5	0.98
	FFPE 36	Positive (Gly12Asp)	71	1,110	1100.2	0.99
	FFPE 71	Positive (Gly32Asp)	27	666	1138.1	0.99
