# Supplementary figures and images for: Development and Validation of Targeted Gene Sequencing Panel Based Companion Diagnostic for Korean Patients with Solid Tumors
Source: Cancers (Basel). 2021 Oct 12;13(20):5112. doi: 10.3390/cancers13205112 (PMC8534153; doi:10.3390/cancers13205112)

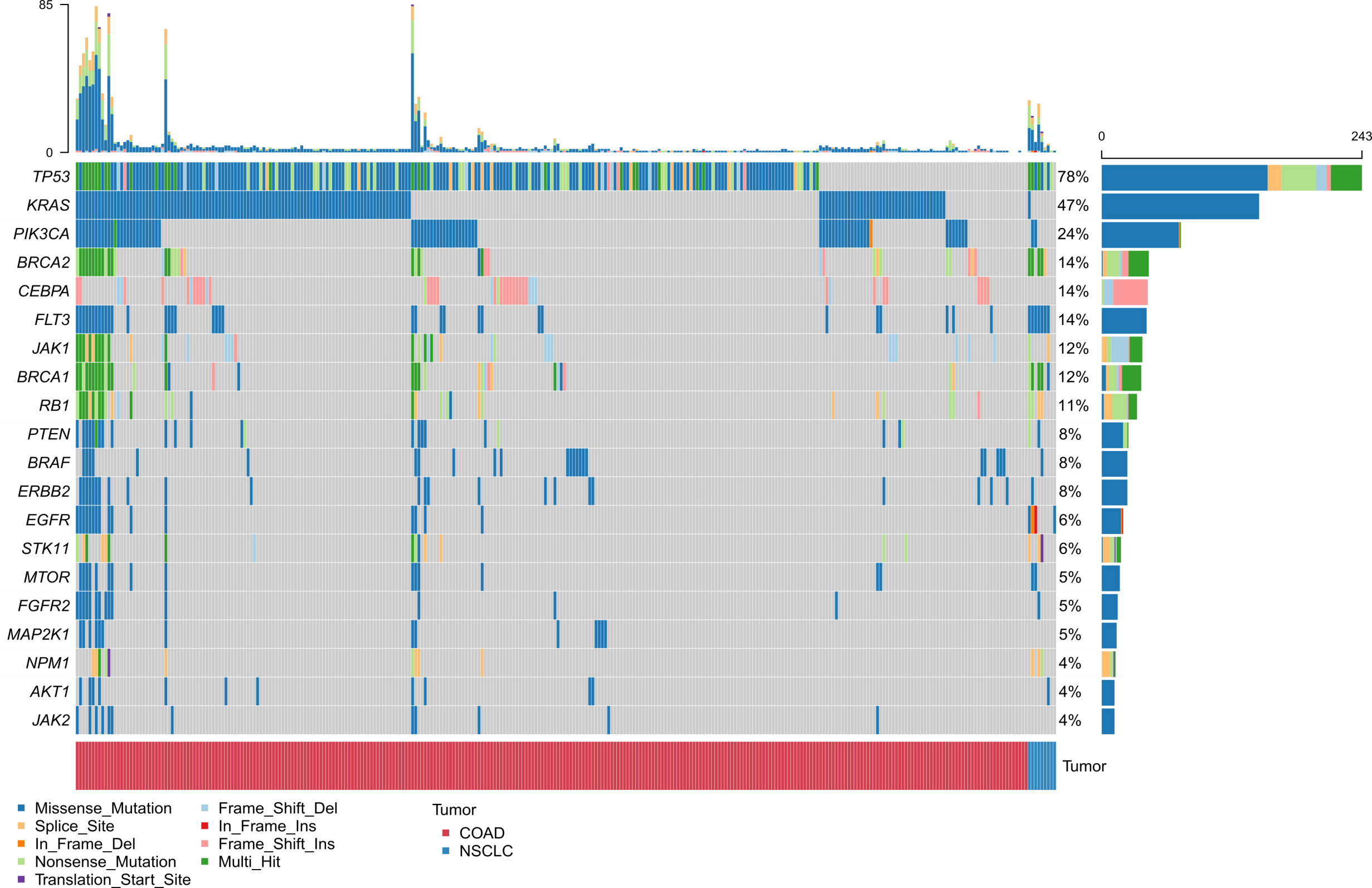

Supplement: Supplementary file 1 [file cancers-13-05112-s001.zip › Supplementary Figure S1.pdf]

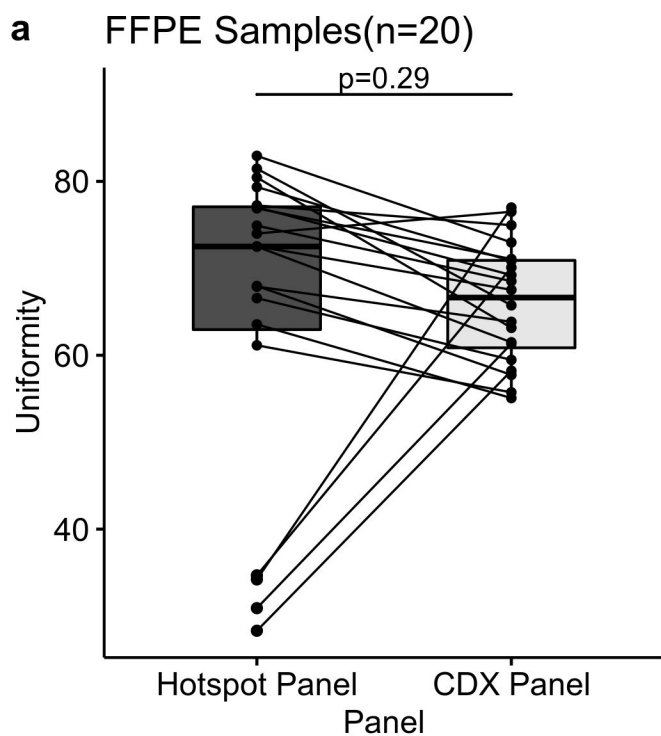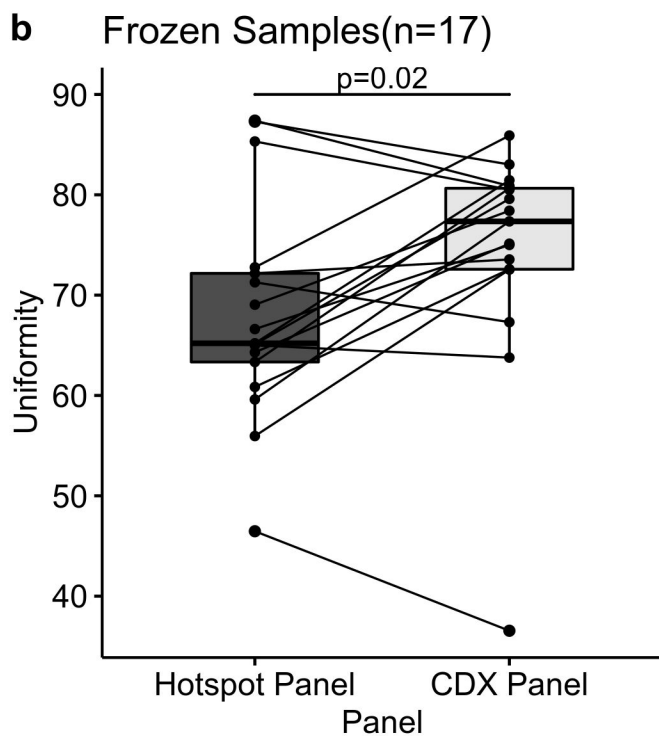

Supplement: Supplementary file 1 [file cancers-13-05112-s001.zip › Supplementary Figure S2.pdf]

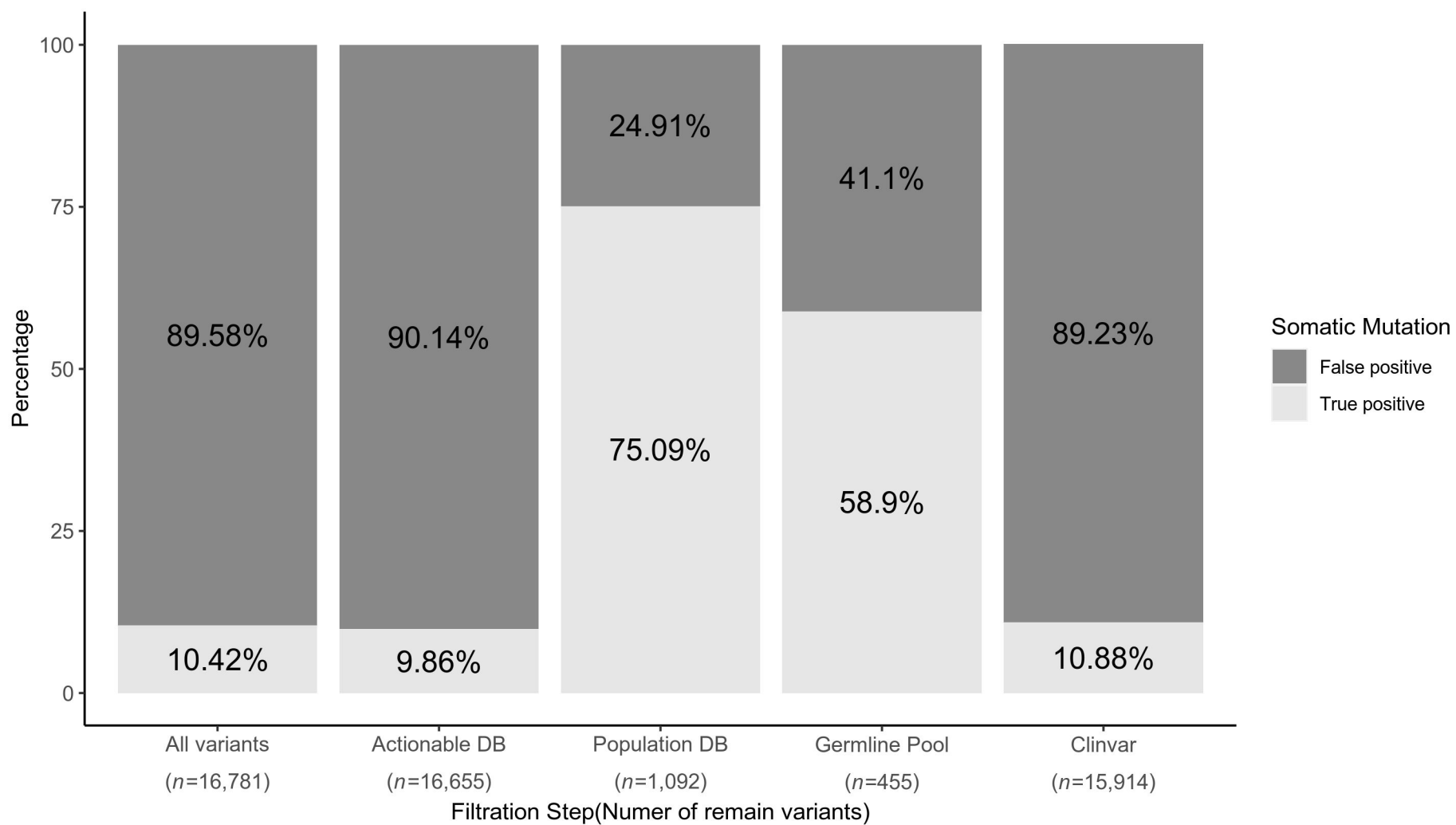

Supplement: Supplementary file 1 [file cancers-13-05112-s001.zip › Supplementary Figure S3.pdf]
